# Detection of a bibenzyl core scaffold in 28 common mangrove and associate species of the Indian Sundarbans: potential signature molecule for mangrove salinity stress acclimation

**DOI:** 10.3389/fpls.2023.1291805

**Published:** 2024-01-16

**Authors:** Bhanumati Sarkar, Hemendra Nath Kotal, Chayan Kumar Giri, Anup Mandal, Nandagopal Hudait, Nithar Ranjan Madhu, Subhajit Saha, Sandip Kumar Basak, Jhimli Sengupta, Krishna Ray

**Affiliations:** ^1^ Department of Botany, Acharya Prafulla Chandra College, Kolkata, West Bengal, India; ^2^ Environmental Biotechnology Group, Department of Botany, West Bengal State University, Kolkata, India; ^3^ Department of Chemistry, West Bengal State University, Kolkata, India; ^4^ Department of Zoology, Acharya Prafulla Chandra College, Kolkata, West Bengal, India; ^5^ Department of Botany, Sarat Centenary College, Dhaniakhali, West Bengal, India

**Keywords:** mangrove, Indian Sundarbans, bibenzyl, hypersalinity, phenylpropanoid pathway, ROS detoxification

## Abstract

Bibenzyl derivatives comprising two benzene rings are secondary plant metabolites with significant therapeutic value. To date, bibenzyl derivatives in the Plant kingdom have been primarily identified in bryophytes, orchids, and *Cannabis sativa*. The metabolic cost investment by plant species for the synthesis of these bioactive secondary metabolites is rationalized as a mechanism of plant defense in response to oxidative stress induced by biotic/abiotic factors. Bibenzyl derivatives are synthesized from core phenylpropanoid biosynthetic pathway offshoots in plant species. Mangrove and mangrove associate species thrive under extreme ecological niches such as a hypersaline intertidal environment through unique adaptive and acclimative characteristics, primarily involving osmotic adjustments followed by oxidative stress abatement. Several primary/secondary bioactive metabolites in mangrove species have been identified as components of salinity stress adaptation/acclimation/mitigation; however, the existence of a bibenzyl scaffold in mangrove species functioning in this context remains unknown. We here report the confirmed detection of a core bibenzyl scaffold from extensive gas chromatography-mass spectrometry and gas chromatography-flame ionization detection analyses of 28 mangrove and mangrove associate species from the Indian Sundarbans. We speculate that the common presence of this bibenzyl core molecule in 28 mangrove and associate species may be related to its synthesis via branches of the phenylpropanoid biosynthetic pathway induced under high salinity, which functions to detoxify reactive oxygen species as a protection for the maintenance of plant metabolic processes. This finding reveals a new eco-physiological functional role of bibenzyls in unique mangrove ecosystem.

## 1 Introduction

Mangrove species and their associates epitomize an inimitable ecosystem at intertidal regions. The ability of the assembly of these heterogeneous species to thrive under an extremely stressful environment is related to their inherent unique adaptive and acclimative traits (Nizam et al., 2022). Mangrove species synthesize a large array of primary and secondary metabolites as a protective response to shield their metabolic processes from the surrounding hypersalinity and other environmental stress factors, which comes at a cost of considerable investment of energy and resources (Wang et al., 2016; Begam et al., 2020; Sudhir et al., 2022). Apart from primary metabolites such as soluble sugars and free amino acids (e.g., proline), the synthesis and accumulation of quaternary ammonium compounds (e.g., glycine betaines), sugar alcohols (e.g., mannitol, ononitol, pinitol, inositol, polyamines, fructans, and trehalose), and several other low-molecular-weight metabolites (compatible solutes) have been extensively reported to facilitate osmotic adjustments in mangrove species, in addition to their salt excretion and ultrafiltration adaptabilities (Munns and Tester, 2008; Slama et al., 2015; Wang et al., 2016; Begam et al., 2020). Intriguingly, several mangrove and mangrove associate species are also known to be valuable bio-resources of several secondary bioactive molecules such as polyphenols, flavonoids, anthocyanins, lignins, triterpenoids, prenylated terpenoids, limonoids, flavonoids, phenolics, tannins, polyisoprenoids, steroids, alkaloids, and saponins, many of which are reported to be of high medicinal value, high antioxidant potential of most of these secondary metabolites being the most common pharmacological activity (Bandaranayake, 1998; Jiang et al., 2018; Zhang et al., 2018; Bibi et al., 2019; Roy and Dutta, 2021; Sudhir et al., 2022; Wu et al., 2022). However, ethnobotanical uses of these mangrove species pose a grave human health risk due to bioaccumulation of potentially toxic elements/heavy metals reported to occur in present day mangroves, a consequence of various environmental pollution and degradation criteria caused by natural and anthropogenic factors, prevailing across mangroves of Indian Sundarbans and Mallorquin Lagoon, Colombian Caribbean, both well-known Ramsar wetland sites (Chowdhury et al., 2021; Chowdhury et al., 2023; Garcés−Ordóñez et al., 2023). The high metabolic costs expended for the synthesis of these bioactive secondary metabolites (known as plant defense contrivances)—apparently derived from the parent core phenylpropanoid biosynthetic pathway and its associated branch points—may be essentially justified by the benefit accrued in mitigating the oxidative stress damage induced under the extreme niche parameters of the mangrove habitat, including high salinity (Dixon and Paiva, 1995; Wang et al., 2016).

**Figure 1:**
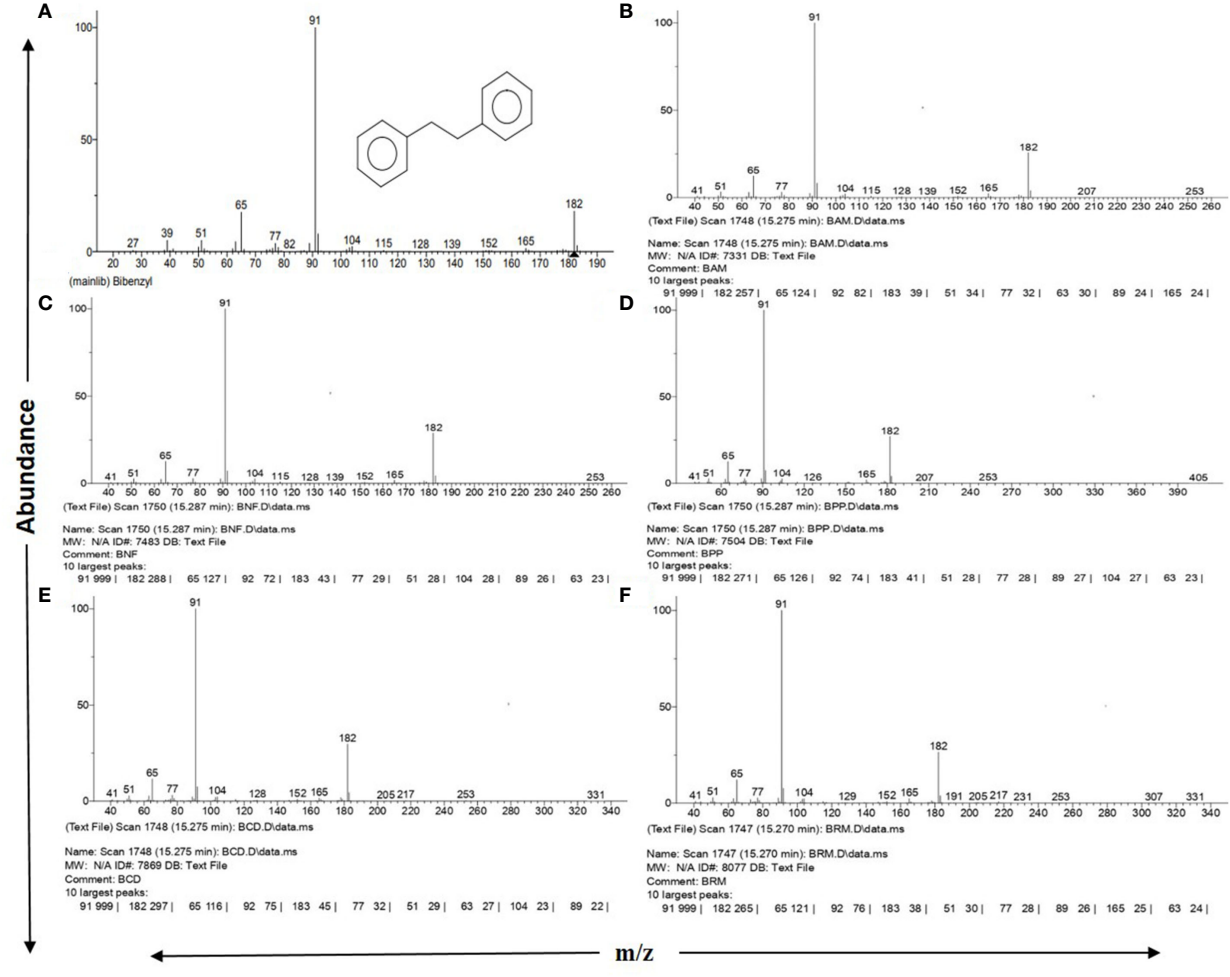
NIST 2.0 generated reports on GC-MS spectra matching with standard Bibenzyl. **(A)** Standard Bibenzyl from NIST mainlib., **(B)** *Avicennia marina* leaf extract, **(C)** *Nypa fruticans* leaf extract, **(D)** *Phoenix paludosa* leaf extract, **(E)** *Ceriops decandra* leaf extract, **(F)** *Rhizophora* sp. leaf extract. All showing 10 largest peaks according to NIST database along with 3 major diagnostic fragment peaks at m/z 65, 91 and 182 with respective abundances.

One group of such secondary metabolites with a common “bibenzyl” core structure has been widely reported in plants, which is considered to possess varying therapeutic attributes (Nandy and Dey, 2020; Ahmad et al., 2022; Boddington et al., 2022; Chen et al., 2022). Bibenzyls have a common parent nucleus consisting of two benzene rings as a derivative of ethane (C6-C2-C6) (**Figure 1A**) with different substituent groups (Zhang et al., 2007; Nandy and Dey, 2020). Bibenzyl compounds are proposed to represent the direct descendants of metabolic branch routes, arisen out of the core phenylpropanoid pathway in all plant types that yield bibenzyl derivatives, including bryophytes (Friederich et al., 1999; Gao et al., 2015), *Cannabis sativa* (Boddington et al., 2022), and orchids (Fritzemeier and Kindl, 1983; Ahmad et al., 2022). Nonetheless, the detailed biosynthetic steps of bibenzyl derivatives for all other plant species that synthesize these compounds have not yet been elucidated.

Under this context, in this study, we detected the bibenzyl core molecule in different parts of 28 mangrove and mangrove associate species from the Indian Sundarbans (**Table 1**). Our repeated gas chromatography (GC)-mass spectrometry (MS) and GC- flame ionization detection (FID) data strongly indicated the presence of a bibenzyl core molecule in almost all the common mangrove and associate species collected from the Indian Sundarbans mangrove ecosystem. Based on these preliminary findings, we hypothesize that the common stress factor of high salinity for all these mangrove species induces enzymes of the phenylpropanoid pathway, including phenylalanine ammonia lyase (PAL) as the very first enzyme of the pathway (Wang et al., 2016), leading to the synthesis and accumulation of bibenzyls in these plants. This specific acclimative feature of mangrove species enables them to considerably detoxify reactive oxygen species (ROS), which can explain the well-known high ROS-scavenging potential of purified bibenzyl compounds of plant origins (Zhang et al., 2007; Cioffi et al., 2011; Kongkatitham et al., 2018). Thus, bibenzyls are implicated here an innate eco-physiological functional trait for mangrove species.

**Table 1:** Presence of Bibenzyl detected via GC-MS in 31 plant parts of 28 mangrove and mangrove associate species of Indian Sundarbans with NIST library spectra match criteria based on NIST algorithm (version NIST 2.0).

Serial No.	Name of mangrove plant species	Name of mangrove associate plant species	Plant part as source	Sampling sites & their location	Surface water salinity range of sampling sites (dSm^-1^)	Sediment salinity range of sampling sites (dSm^-1^)	Compound as closest match in NIST database	MF (Match factor)	RMF (Reverse Match Factor)	Probability (%)	RT (Retention time in minutes)	Peak area%
1		*Acanthus ilicifolius*	Leaf	Ramganga 21°47’33.40”N, 88°22’54.89”E	26.936-40.533	4.505-6.606	Bibenzyl	930	937	85.30	15.281	0.271
2		*Acanthus ilicifolius*	Root	Ramganga 21°47’33.40”N, 88°22’54.89”E	26.936-40.533	4.505-6.606	Bibenzyl	941	945	91.40	15.287	0.141
3		*Brownlowia tersa*	Leaf	Bhagabatpur 21°43’11.92”N, 88°18’34.50”E	26.639-40.992	4.417-9.214	Bibenzyl	932	936	90.50	15.293	0.018
4	*Phoenix paludosa*		Fruit	Satyadaspur 21°44’4.72”N, 88°23’19.95”E	26.936-40.981	1.937-5.35	Bibenzyl	924	933	90.00	15.287	0.253
5	*Phoneix paludosa*		Leaf	Satyadaspur 21°44’4.72”N, 88°23’19.95”E	26.936-40.981	1.937-5.35	Bibenzyl	938	945	90.80	15.287	0.158
6		*Suaeda* sp.	Leaf	Daxinlaxminarayanpur 21°45’43.90”N, 88°20’39.91”E	21.91-40.251	4.449-4.854	Bibenzyl	937	940	91.80	15.293	0.027
7	*Xylocarpus* sp.		Fruit	Krishnadaspur 21°42’42.00”N, 88°24’20.57”E	22.237-39.86	6.128-7.905	Bibenzyl	923	932	89.10	15.287	0.127
8	*Avicennia marina*		Leaf	Durbachoti 21°51’15.09”N, 88°18’45.15”E	17.179-33.972	4.23-5.121	Bibenzyl	915	925	88.60	15.275	0.163
9	*Aegiceras corniculatum*		Leaf	Ramganga 21°47’33.40”N, 88°22’54.89”E	26.936-40.533	4.505-6.606	Bibenzyl	939	943	89.60	15.275	0.137
10	*Heritiera fomes*		Leaf	Bhagabatpur 21°43’11.92”N, 88°18’34.50”E	26.639-40.992	4.417-9.214	Bibenzyl	910	914	82.70	15.287	0.173
11	*Bruguiera cylindrica*		Leaf	Daxinshibganj 21°46’37.14”N, 88°22’36.23”E	15.5-39.348	5.569-8.453	Bibenzyl	769	926	85.80	15.27	0.007
12	*Bruguiera gymnorrhiza*		Leaf	Durbachoti 21°51’15.09”N, 88°18’45.15”E	17.179-33.972	4.23-5.121	Bibenzyl	939	947	91.90	15.281	0.190
13	*Bruguiera gymnorrhiza*		Fruit	Durbachoti 21°51’15.09”N, 88°18’45.15”E	17.179-33.972	4.23-5.121	Bibenzyl	713	912	56.30	15.130	0.125
14	*Bruguiera parviflora*		Leaf	Ramganga 21°47’33.40”N, 88°22’54.89”E	26.936-40.533	4.505-6.606	Bibenzyl	903	919	83.80	15.27	0.131
15	*Sonneratia apetala*		Young fruits	Rakhalpur 21°45’7.73”N, 88°27’46.48”E	29.747-40.432	7.655-12.345	Bibenzyl	932	936	89.80	15.281	0.180
16	*Sonneratia caseolaris*		Leaf	Khetramohanpur 21°45’42.98”N, 88°21’0.41”E	21.93-40.128	8.133-9.281	Bibenzyl	932	944	91.60	15.281	0.069
17	*Excoecaria agallocha*		Leaf	Debichak 21°50’21.62”N, 88°22’15.71”E	15.447-22	2.088-2.928	Bibenzyl	920	928	90.50	15.287	0.144
18		*Derris trifoliata*	Leaf	Shiberghat 21°49’21.68”N 88°21’20.68”E	25.892-36.003	8.959-10.614	Bibenzyl	932	940	90.40	15.281	0.294
19	*Nypa fruticans*		Young leaf	Bhagabatpur 21°43’11.92”N, 88°18’34.50”E	26.639-40.992	4.417-9.214	Bibenzyl	916	924	88.80	15.287	0.115
20	*Ceriops tagal*		Leaf	Atherogaji 21°50’49.47”N, 88°23’15.96”E	23.031-40.533	4.203-6.224	Bibenzyl	915	942	90.70	15.27	0.127
21	*Ceriops decandra*		Leaf	Atherogaji 21°50’49.47”N, 88°23’15.96”E	23.031-40.533	4.203-6.224	Bibenzyl	913	937	30.10	15.275	0.073
22		*Acrostichum aureum*	Leaf	Bhagabatpur 21°43’11.92”N, 88°18’34.50”E	26.639-40.992	4.417-9.214	Bibenzyl	692	912	61.80	15.130	0.152
23		*Merope angulata*	Leaf	Daxinshibganj 21°46’37.14”N, 88°22’36.23”E	15.5-39.348	5.569-8.453	Bibenzyl	664	907	50.60	15.136	0.101
24	*Kandelia candel*		Leaf	Ramganga 21°47’33.40”N, 88°22’54.89”E	26.936-40.533	4.505-6.606	Bibenzyl	602	899	24.60	15.141	0.045
25		*Thespesia populnea*	Leaf	Bhagabatpur 21°43’11.92”N, 88°18’34.50”E	26.639-40.992	4.417-9.214	Bibenzyl	731	909	64.60	15.130	0.141
26	*Lumnitzera racemosa*		Leaf	Chotorakkhoskhali 21°44’55.48”N, 88°23’16.39”E	15.921-37.272	6.554-9.233	Bibenzyl	775	949	83.40	15.130	0.200
27		*Caesalpinia bonduc*	Leaf	Banashyamnagar 21°47’15.23”N, 88°23’36.04”E	30.187-39.321	4.773-8.47	Bibenzyl	807	919	77.80	15.124	0.094
28		*Caesalpinia crista*	Leaf	Brajoballavpur 21°42’48.96”N, 88°23’42.66”E	30.334-40.543	6.788-10.345	Bibenzyl	915	935	90.00	15.275	0.091
29		*Acanthus volubilis*	Leaf	Rakhalpur 21°45’7.73”N, 88°27’46.48”E	29.747-40.432	7.655-12.345	Bibenzyl	760	939	72.20	15.118	0.076
30	*Rhizophora* sp.		Leaf	Shiberghat 21°49’21.68”N 88°21’20.68”E	25.892-36.003	8.959-10.614	Bibenzyl	906	936	89.60	15.27	0.021
31		*Dalbergia spinosa*	Leaf	Durgagobindapur 21°48’9.12”N, 88°21’30.67”E	20.897-37.558	7.765-10.318	Bibenzyl	899	931	90.60	15.276	0.029

## 2 Materials and methods

### 2.1 Collection of plant samples

**Figure 2:**
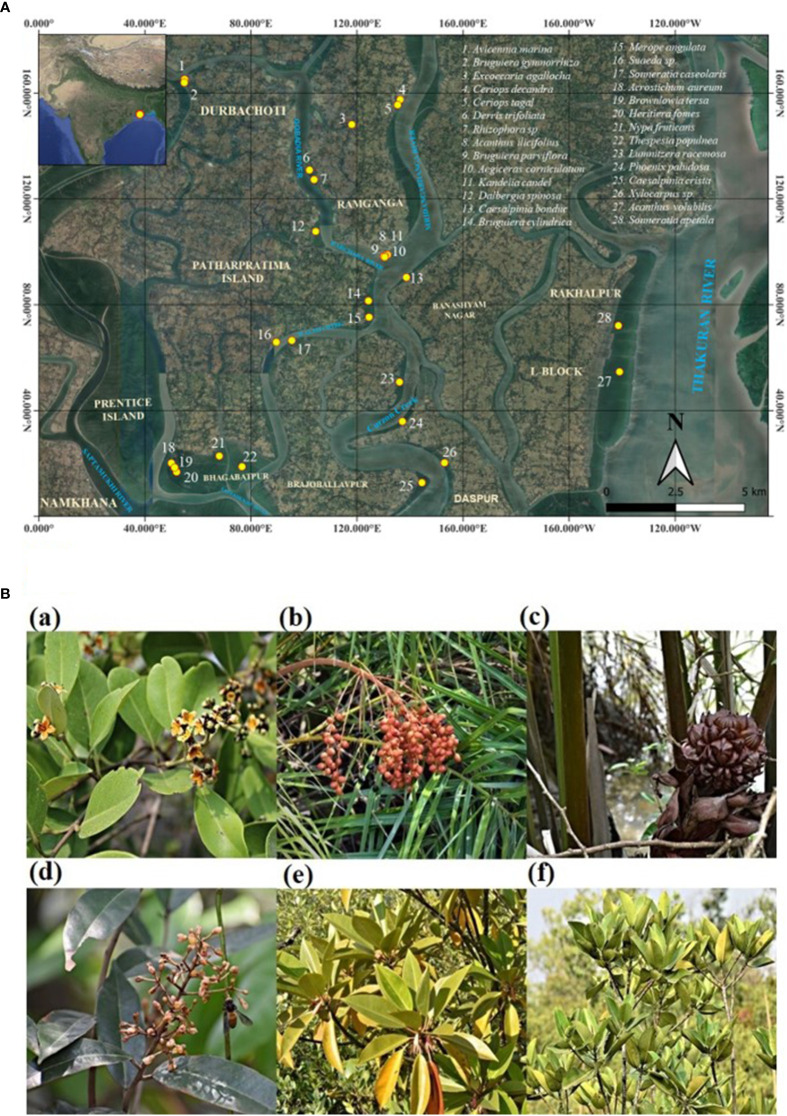
**(A)** A comprehensive map detailing the specific sampling and distribution locations of every mangrove and mangrove associate species collected in this study across Indian Sundarbans. **(B)** Some representative mangrove and mangrove associate species of Indian Sundarbans where bibenzyl scaffold is detected. (a) *Avicennia marina* (b) *Phoenix paludosa* (c) *Nypa fruticans* (d) *Brownlowia tersa* (e) *Bruguiera gymnorrhiza* (f) *Rhizophora* sp.

Fresh leaves, young roots, young twigs, and immature fruit samples of 28 mangrove and associate species (*Avicennia marina*, *Acanthus ilicifolius*, *Phoenix paludosa*, *Nypa fruticans*, *Suaeda* sp., *Aegiceras corniculatum*, *Merope angulata*, *Acrostichum aureum*, *Acanthus volubilis*, *Bruguiera gymnorrhiza*, *Bruguiera cylindrica*, *Bruguiera parviflora*, *Caesalpinia bonduc*, *Caesalpinia crista*, *Dalbergia spinosa*, *Ceriops decandra*, *Ceriops tagal*, *Derris trifoliata*, *Excoecaria agallocha*, *Heritiera fomes*, *Kandelia candel*, *Lumnitzera racemosa*, *Rhizophora* sp., *Sonneratia apetala*, *Sonneratia caseolaris*, *Thespesia populnea*, *Xylocarpus* sp., and *Brownlowia tersa*) were collected from different villages of the Patharpratima block of the Indian Sundarbans and Bhagabatpur protected mangroves under the Sundarbans Biosphere Reserve (**Figures 2A, B**; **Table 1**). The location coordinates of all the sampling sites, the surface water salinity range and the salinity range of sediments are provided in **Table 1**.

### 2.2 Preparation of plant extracts for downstream analyses

The freshly collected leaves and other plant parts were thoroughly washed in running tap water and rinsed in deionized water two to three times. The washed samples were then cut into small pieces, shade-dried at room temperature for 2–3 h, and ground into a powder using an oven-dried mortar and pestle. The ground plant tissues (1 g) were macerated with 3–5 mL of high-performance liquid chromatography (HPLC)-grade methanol (Merck) proportionately. The mixture was filtered with Whatman No. 1 filter paper and the filtrate was concentrated using a rotary evaporator (EYELA N-1200A) at 37°C for 30–40 min and placed in a 40°C water bath. The concentrated crude methanolic extract collected from the rotary evaporator was transferred to a separation funnel with 2–3 mL of dichloromethane (DCM), vigorously shaken, and allowed to stand for 15–20 min for separation. The DCM phase was separated out with chlorophyll. Both the methanolic and DCM phases were collected separately and the methanolic phase was again repeatedly extracted with a DCM:brine (1:1) (saturated NaCl) mixture. The final methanol-aqueous phase fraction was collected and passed through a sodium sulfate column to remove the water. Finally, the solvent was completely removed under reduced pressure by a rotary evaporator in a 40°C water bath for 20 min. The dried fraction was re-dissolved in 1 mL of HPLC-grade methanol, collected into GC vials, and stored at 4°C for further GC-MS and GC-FID analyses.

### 2.3 GC-MS analysis

GC-MS analyses were carried out using the Agilent 7890B series GC and 5977A mass spectrometer equipped with a fused silica column, packed with an HP-5MS capillary column (30 m long  ×  250 μm diameter  ×  0.25 μm thick). Pure helium gas (99.99%) was used as the carrier gas at a constant flow rate of 1.2 mL/min. One microliter of the sample was injected (split ratio 10:1) and the injector temperature was maintained at 250°C. The column oven temperature was set to 40°C for 2 min, raised at 10°C/min up to 230°C, and the final temperature was increased up to 300°C at increments of 10°C/min for 8.5 min. The total run time was 33 min. For spectral detection, fragments ranging from 40 to 600 m/z of phytochemicals present in the test samples were identified based on comparison of their retention time (RT; min), peak abundance, peak height, and mass spectral patterns according to the spectral database of authentic standard compounds stored in the National Institute of Standards and Technology (NIST) library (version 2.0) based on the NIST internal algorithm (NIST version 2.0; https://chemdata.nist.gov/). All the plant extracts were run with three biological replicates with a concomitant blank run for the methanol solvent.

### 2.4 GC-FID analysis

An Agilent Model 6890N gas chromatograph was utilized for GC-FID analyses. One milliliter of the prepared solutions was placed into an autosampler vial for analysis and separation was achieved on an HP-5MS column (30 m long  ×  250 μm diameter  ×  0.25 μm thick) using three concentrations of bibenzyl internal standard (99% pure; Sigma-Aldrich) at concentrations of 0.50, 1, and 2 mg/L in methanol. Ultra-high purity (99.999%) helium was chosen as the carrier gas with a flow rate of 1.0 mL/min. The flame ionization detector was sustained at a temperature of 320°C and the inlet temperature was maintained at 250°C in splitless mode (10:1). One microliter of each sample was injected. An isothermally programmed oven was adjusted to an initial temperature of 60°C held for 2 min, increased to 100°C at 20°C/min and held for 2 min, then increased to 310°C at 40°C/min and held for 14 min; helium was utilized as the auxiliary make-up gas for the detector at a flow rate of 30 mL/min. The total run time was 25.25 min. The presence of the bibenzyl core compound in plant samples was confirmed by comparing the peak abundance and RT (min) based on Agilent MassHunter-generated chromatogram reports with those of the authentic bibenzyl standards run simultaneously, and the peak areas were measured to quantify the bibenzyl content in the test samples on the basis of the peaks for the concurrently run standards. The plant extracts were run in three biological replicates, each time including a parallel run for the authentic bibenzyl standard and a blank for the methanol solvent.

## 3 Results

### 3.1 GC-MS data analyses

GC-MS detected the presence of a core bibenzyl molecule in the methanolic extracts of 31 different plant parts belonging to 28 species of true mangrove and mangrove associates growing in the same niche as a unique plant community in the Indian Sundarbans. The generated mass spectral data were compared against the NIST library spectra to obtain the Match Factor (Match), Reverse Match Factor (R. Match), and Probability (%) values to create a hit list of the compounds detected by GC-MS (**Table 1**). In almost all the samples, the Match and R. Match scores were >900 with the probability ranging from 80% to 91%, proving this “bibenzyl” identification as an “Excellent Match” based on the NIST algorithm. The first three hits in the hit list were ultimately identified as “bibenzyl” for all 31 plant parts examined (**Table 1**). The RT of this “bibenzyl” molecule was observed to be ~15 min for all test samples (**Table 1**) in the GC-MS analyses. From the GC-MS study the peak area percentage ranged from 0.007% to 0.294% across all the plant samples studied (**Table 1**) where the referred peak at this typical RT was found to match with “bibenzyl” molecule based on NIST internal algorithm (NIST Version 2.0).

The mass spectral pattern generated for all 31 tested plant parts and their replicates distinctively matched with NIST Mass Spectrometry Data Center information on mass spectra identification of molecules, where the “bibenzyl” molecule with a molecular weight of 182.26 g/mol (https://pubchem.ncbi.nlm.nih.gov/compound/Bibenzyl) was stated to show diagnostically three major peaks of highest abundance in GC-MS: m/z 91 as the most abundant peak, followed by m/z 182 and m/z 65 as second and third highest abundant peaks, respectively (https://pubchem.ncbi.nlm.nih.gov; http://www.nist.gov/srd/nist1a.cfm) (**Figures 1A–F**).

### 3.2 GC-FID data analyses

**Figure 3:**
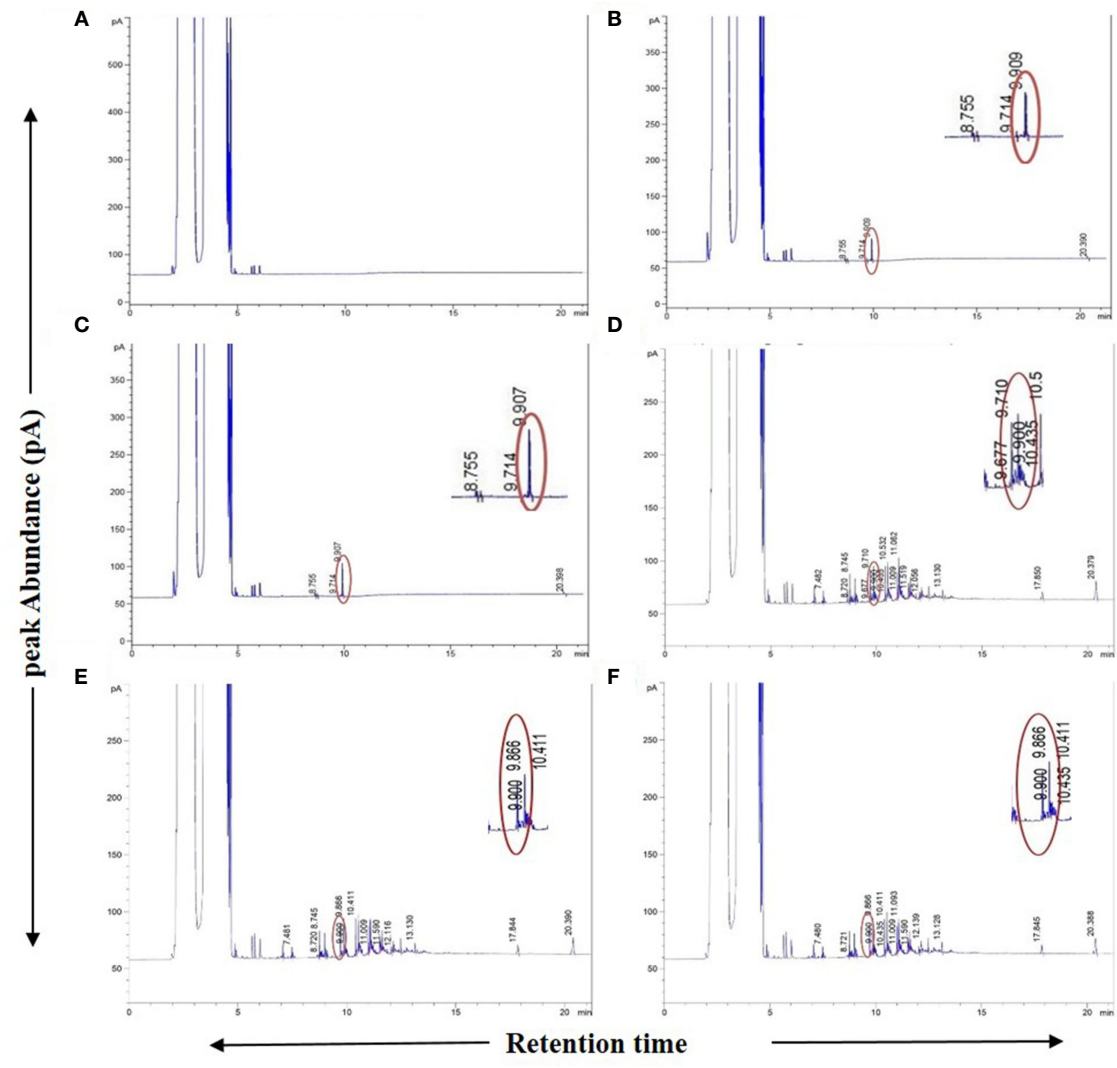
GC-FID spectra of Agilent MassHunter database generated chromatogram reports. **(A)** Blank (only solvent), **(B)** 1 ppm (1µg/ml) standard Bibenzyl, **(C)** 2 ppm (1µg/ml) standard Bibenzyl, **(D)** *Avicennia marina* leaf extract, **(E)** *Phoenix paludosa* leaf extract, **(F)** *Nypa fruticans* leaf extract. Chromatograms showing sample leaf extract peak at 9.9 min. retention time matching with standard Bibenzyl peak in concurrent run at retention time of ~9.909 min. with respective peak abundances.

As an additional authentication step, GC-FID analyses were carried out on the plant samples in which the presence of “bibenzyl” was detected by GC-MS with an identical RT for the peak of the authentic bibenzyl standard (~9.90 min) from the Agilent MassHunter database. This compound was also evident from the GC-FID chromatograms for these plant samples (**Figures 3A–F**). The difference in RT of bibenzyl between the two different individual GC machines used for GC-MS (RT ~15 min) and GC-FID (RT ~9.90 min) could be attributed to the different total run times and differently programmed oven temperatures used for the two different models, although the column model used in both cases was the same. Despite the use of two different gas chromatograph machines with varying run conditions for GC-MS and GC-FID, the presence of the “bibenzyl” core scaffold in 31 plant extracts and their biological replicates belonging to 28 mangrove and mangrove associate species could be unequivocally established from both analyses (**Table 1**; **Figures 1A–F**, **3A–F**; **Supplementary Figures 4A, B**).

The peak areas calculated at a typical RT of ~9.90 min for different concentrations of the standard bibenzyl compound were utilized to construct a standard curve to quantify the amount of bibenzyl in fresh plant tissue from the respective GC-FID chromatograms (**Supplementary Figure 4B**). The quantified content of bibenzyl ranged from approximately 0.2 to 1.7 µg/g of fresh tissue. This quantified bibenzyl content in mangrove plant extracts is much lower than that reported in other plant species. For erianin and gigantol bibenzyl derivatives from *Dendrobium officinale* plant parts, quantified via liquid chromatography-MS, it was reported as 2.63 ± 0.69 and 37.01 ± 2.16 µg/g, respectively in root tissues whereas 0.61± 0.01 and 22.67 ± 0.15 µg/g respectively was found in basal stem tissues (Adejobi et al., 2021). No bibenzyl compounds could be detected from leaf tissues in this study (Adejobi et al., 2021). For moscatilin, gigantol, crepidatin, and chrysotoxin bibenzyl derivatives from the dried stems of *Dendrobium* spp. (33 species) from Thailand, detected by HPLC, the total bibenzyl derivative content ranged from the highest level of 7.36 ± 0.50 mg/g dry weight in *D. fimbriatum* to the lowest level of 0.07 ± 0.00 mg/g dry weight in *D. christyanum* among all 33 species (Choonong et al., 2019). The total bibenzyl content from the dried roots, pseudobulbs, and leaves of *Dendrobium sinense*, measured via spectrophotometry using bibenzyl gigantol as a standard was reported to be 1.31%, 0.62%, and 0.72% respectively of the dry weight of roots, pseudobulbs, and leaves (Chen et al., 2022).

## 4 Discussion

The main finding of a bibenzyl molecule detected in 28 mangrove species through this extensive GC-MS and GC-FID study initially seemed to be very intriguing. To date, among plants, bibenzyl derivatives have mainly been reported from some bryophytes, orchids, and *Cannabis sativa*, and some of these derivatives are well established for several clinical parameters, mainly based on their antimicrobial and cytotoxic potential (Nandy and Dey, 2020; Ahmad et al., 2022; Boddington et al., 2022; Chen et al., 2022). Several mangrove and mangrove associate species are often reported to possess bioactive secondary metabolite molecules of high curative, preventive, and pharmaceutical importance in medicinal practices (Bandaranayake, 1998; Jiang et al., 2018; Zhang et al., 2018; Bibi et al., 2019; Roy and Dutta, 2021; Sudhir et al., 2022; Wu et al., 2022). Nevertheless, to our best knowledge, the presence of bibenzyls has not yet been referred to in this context. Under this backdrop, it seemed challenging to interpret and rationalize the presence of bibenzyl molecules in 28 mangrove and mangrove associate species collected from the Indian Sundarbans without downstream purification of the actual bibenzyl derivatives detected. However, the repeated identification of the bibenzyl cores in such a high number of mangrove and mangrove associate species (28 species) with almost 95–100% reproducibility in the results irrevocably establishes the bibenzyl core molecule as a product of some yet-to-be-identified secondary metabolic pathway existing in these eco-physiologically enigmatic mangrove and mangrove associate species. As these findings are preliminary and we are yet to purify the “bibenzyl” derivative compounds from these mangrove and associate species, the ubiquitous presence of bibenzyl scaffold in all these 28 species, strengthens our hypothesis to justify that all these studied mangrove and associate species invest their energy in synthesizing this common secondary metabolite “bibenzyl”, because the presence of this metabolite could be related to the ROS-scavenging potential of the bibenzyl molecule, which is desperately needed by the mangrove and mangrove associate species to mitigate the salinity induced oxidative stress to survive in hypersaline mangrove niche. To validate our hypothesis to establish “bibenzyl” as a novel eco-physiological trait of mangrove species, hitherto unknown in existing literature, we carried out this extensive study of detection of the presence of bibenzyl core scaffold for 28 different mangroves and related plant species.

Several researchers have demonstrated bibenzyl biogenesis in plant systems as a by-product of branches of the core phenylpropanoid biosynthetic pathway. A well-known bibenzyl derivative (3,3’,5-trihydroxybibenzyl) identified from the bulb tissues of the orchids *Barlia longibracteata* and *Orchis* spp. was first validated to be a physiological intermediate of an offshoot of the phenylpropanoid pathway, with l-phenylalanine as a precursor and various hydroxycinnamic acids as intermediary substrates. This study conclusively established the origin of at least one of the aromatic rings in the trihydroxybibenzyl molecule derived via phenylpropanoid biosynthesis based on feeding experiment results (Fritzemeier and Kindl, 1983). A correlation between upregulated expression of the several intermediate enzymes of the phenylpropanoid pathway, including the starting enzyme PAL, in the roots, leaves, and several other plant parts with concurrent abundance of bibenzyls in the orchid *Arundina graminifolia* indirectly indicated the interrelationship between these molecules (Ahmad et al., 2022). The biosynthetic route of cyclic bis(bibenzyl) marchantin A, a derivative of the core bibenzyl from the bryophyte *Marchantia polymorpha*, was also demonstrated to originate from the benzene ring of l-phenylalanine via trans-cinnamic acid and *p*-coumaric acid. The researchers validated the biosynthesis of the bibenzyl monomers to constitute the marchantin A molecule via a unique branch of the core phenylpropanoid biosynthesis pathway (Friederich et al., 1999). Another bryophyte, *Plagiochasma appendiculatum*, was also successfully demonstrated to synthesize bisbibenzyls via a special branch of the core phenylpropanoid synthesis pathway, originating from l-phenylalanine and taking a diversion at *p*-coumaric acid to dihydro-*p*-coumaric acid, leading to the formation of dihydro-*p*-coumaryl coenzyme A (CoA) and other intermediates (Gao et al., 2015). A recent comprehensive study from *Cannabis sativa* explicitly proved the origin of the two aromatic rings of the bibenzyl scaffold derived from the core phenylpropanoid pathway via hydroxycinnamic acids becoming esterified to acyl-CoA to form dihydro-CoA derivatives and consequent condensation with malonyl-CoA to yield bibenzyl scaffolds (Boddington et al., 2022).

Thus, the detection of a bibenzyl scaffold from 28 mangrove and mangrove associate species of the Indian Sundarbans may be rationalized in light of the physiological adaptive convergence of mangrove species, where the primary acclimations are centered on osmotic adjustments and the secondary acclimative process controls oxidative stress-induced damage from the high-salinity environment (Nijam et al., 2022). The phenylpropanoid biosynthetic pathway that provides substrates for many secondary metabolites, including bibenzyls, is an integral component of this oxidative stress mitigation process. This explains the common presence of the bibenzyl scaffold in all 28 mangrove species studied. This interpretation finds stronger support from a study showing significant upregulation of enzymes of the phenylpropanoid pathway that were strongly induced under hypersalinity (500 mM NaCl) in *Kandelia candel*, a woody true mangrove species, demonstrated via transcriptomic analyses, enzymatic activity analyses, and succeeding “phenylpropanoid” accumulation with potential to scavenge ROS (Wang et al., 2016). Several reports corroborate the *in vitro* ROS detoxification attribute of purified biogenic bibenzyl derivatives, including from the orchid *Dendrobium parishii* (Kongkatitham et al., 2018); the aerial parts of *Notholaena nivea* Desv. (Pteridaceae) (Cioffi et al., 2011); purified moscatilin, gigantol, crepidatin, and chrysotoxin bibenzyl derivatives from *Dendrobium* spp. (Choonong et al., 2019); and purified bibenzyls, nobilin D and nobilin E, from the orchid *Dendrobium nobile* (Zhang et al., 2007). This ROS detoxification potential of ‘bibenzyl’ scaffold becomes more important for mangrove associate species, that grow luxuriously in the same mangrove habitats of Indian Sundarbans along with conventionally known true mangrove species irrespective of their habits like herb, shrub or tree. True mangrove species possess typical adaptive features like pneumatophores, vivipary, cryptovivipary etc., which are lacking in these semi-mangrove plants (mangrove associate species). Despite the absence of true mangrove adaptive features, these semi-mangrove plants (mangrove associate species) have the potential to survive the hypersalinity of the mangrove niches because of their acclimative osmotic adjustment potentials by synthesizing several compatible solutes (Begam et al., 2020). Almost all the mangrove associate species we have referred in this study (**Table 1**) are known to synthesize one or more number of compatible solutes, helping them to acclimate with the hypersalinity of mangrove niches by osmotic adjustment, notwithstanding the possession of adaptive features of true mangroves (Begam et al., 2020). This study establishes the presence of another bioactive secondary molecular scaffold of “bibenzyl” being common to all these mangrove associate species, which might confer to these species additional salinity induced ROS scavenging potential via antioxidant activities described for this molecule by earlier researchers (Zhang et al., 2007; Cioffi et al., 2011; Kongkatitham et al., 2018; Choonong et al., 2019). Thus this study signifies “bibenzyl” scaffold, like other secondary metabolites, might be a potential acclimative response that is especially beneficial for mangrove associate species.

In summary, this study confirmed detection of a bibenzyl scaffold from 28 mangrove and mangrove associate species of the Indian Sundarbans growing in mangrove niches where the prevailing conductivity of the sediment and riverine water is ~5–12 dS/m and ~32–40 dS/m (**Table 1**) respectively, during major part of the year (Chowdhury et al., 2019; Begam et al., 2020). We propose bibenzyl as another signature molecule of salinity acclimation in mangroves, with potential for detoxification of salinity-induced ROS, thereby shielding the photosynthetic apparatus and other cell organelles and protecting function of the downstream metabolic processes, similar to the effects of compatible solutes/osmolytes. To our knowledge, this is the first report to reveal the presence of the secondary metabolite bibenzyl in mangroves thought to have an eco-physiological functional role towards salinity acclimation. However, purification of the bibenzyl derivatives from mangrove species is yet to be carried out, along with validation of its biosynthetic steps. This preliminary study should attract further research interest on mangrove eco-physiological functional traits, especially with regard to the mechanisms underlying salinity stress acclimation, amid a unique ecological environment. Bibenzyl seems a potential signature molecule for stress alleviation of mangrove species adapted to hypersaline ecology.

## Data availability statement

The original contributions presented in the study are included in the article/**Supplementary Material**. Further inquiries can be directed to the corresponding author.

## Author contributions

BS: Data curation, Formal analysis, Investigation, Methodology, Writing – original draft. HK: Data curation, Formal analysis, Investigation, Methodology, Writing – original draft, Writing – review & editing. CG: Data curation, Investigation, Methodology, Writing – original draft. AM: Data curation, Investigation, Methodology, Writing – review & editing. NH: Methodology, Writing – review & editing. NM: Investigation, Resources, Writing – review & editing. SS: Data curation, Investigation, Methodology, Writing – original draft. SB: Funding acquisition, Resources, Supervision, Writing – review & editing, Validation. JS: Methodology, Writing – review & editing. KR: Conceptualization, Formal analysis, Funding acquisition, Project administration, Resources, Supervision, Validation, Writing – original draft, Writing – review & editing.

## Supplementary material

Supplementary Figure 4
**(A)** Box-whisker plot representing GC-MS spectra matching criteria with standard Bibenzyl in 28 species of mangrove and mangrove associates of Indian Sundarbans based on NIST database algorithm (NIST 2.0). Match factor (MF), Reverse match factor (RMF) are in scale of 1000 (left Y axis) and probability% is in scale of 100 in right Y axis, n=31. **(B)** GC-FID based quantitative analysis of Bibenzyl in 9 common mangrove species of Indian Sundarbans from a representative run.
