# Supplementary figures and images for: Detection of a bibenzyl core scaffold in 28 common mangrove and associate species of the Indian Sundarbans: potential signature molecule for mangrove salinity stress acclimation
Source: Front Plant Sci. 2024 Jan 16;14:1291805. doi: 10.3389/fpls.2023.1291805 (PMC10824835; doi:10.3389/fpls.2023.1291805)

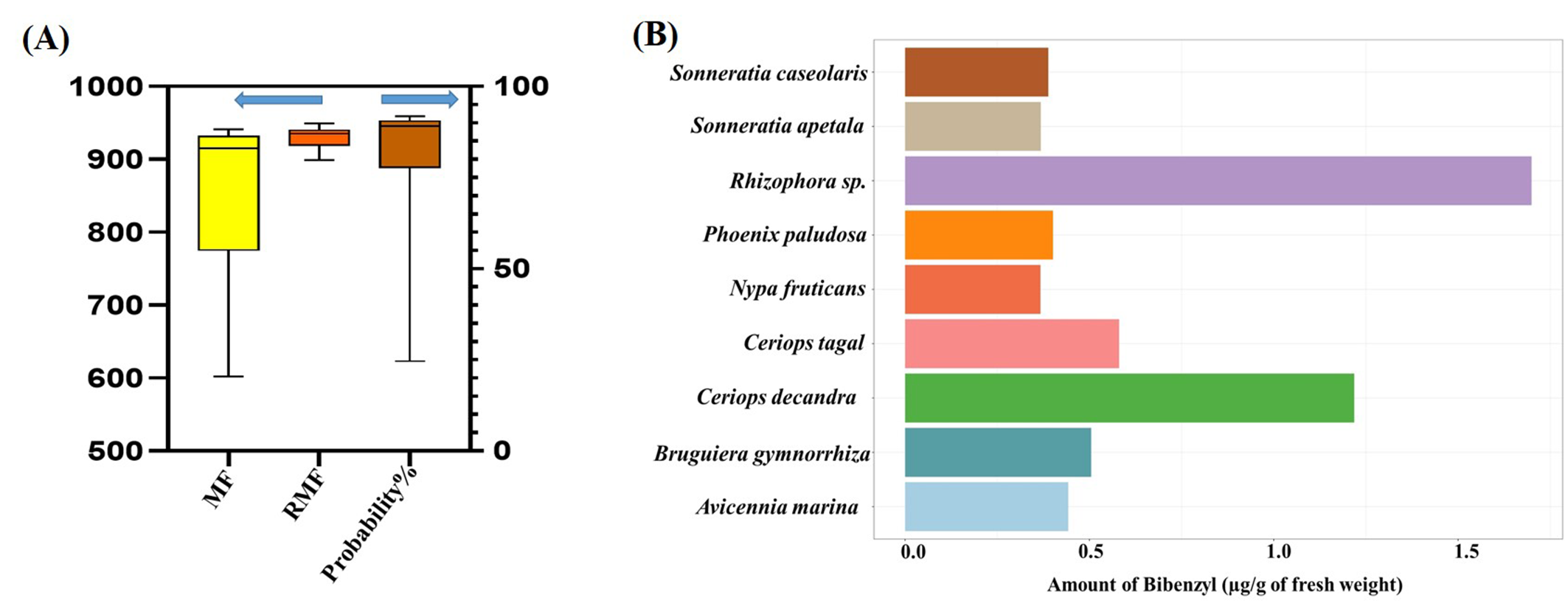

Supplement: Supplementary Figure 4 — (A) Box-whisker plot representing GC-MS spectra matching criteria with standard Bibenzyl in 28 species of mangrove and mangrove associates of Indian Sundarbans based on NIST database algorithm (NIST 2.0). Match factor (MF), Reverse match factor (RMF) are in scale of 1000 (left Y axis) and probability% is in scale of 100 in right Y axis, n=31. (B) GC-FID based quantitative analysis of Bibenzyl in 9 common mangrove species of Indian Sundarbans from a representative run. [file Image_1.tif]
